# Lipid-based nanoparticles for drug delivery in Parkinson’s disease

**DOI:** 10.1515/tnsci-2022-0359

**Published:** 2024-12-03

**Authors:** Han Cai, Dong Liu, Wei-Wei Xue, Liya Ma, Hai-Tao Xie, Ke Ning

**Affiliations:** Guangdong Celconta Biotechnology Co., Ltd, 9 Xincheng Road, Songshan Lake Park, Dongguan, Guangdong, PR China; School of Biological Engineering, Dalian Polytechnic University, Dalian, 116034, China; Sheffield Institute of Translational Neuroscience (SITraN), University of Sheffield, Sheffield, United Kingdom

**Keywords:** Parkinson’s disease, lipid-based nanoparticles, blood‒brain barrier, drug delivery, nanomedicine

## Abstract

Parkinson’s disease (PD) is a neurodegenerative disorder that predominantly affects dopaminergic neurons in the substantia nigra and ventral tegmental area, resulting in symptoms such as tremors, muscle rigidity, bradykinesia, and potential cognitive and affective disturbances. The effective delivery of pharmacological agents to the central nervous system is hindered by various factors, including the restrictive properties of the blood‒brain barrier and blood‒spinal cord barrier, as well as the physicochemical characteristics of the drugs. Traditional drug delivery methods may not provide the therapeutic concentrations necessary for functional restoration in PD patients. However, lipid-based nanoparticles (NPs) offer new possibilities for enhancing the bioavailability of established treatment regimens and developing innovative therapies that can modify the course of the disease. This review provides a concise overview of recent advances in lipid-based NP strategies aimed at mitigating specific pathological mechanisms relevant to PD progression. This study also explores the potential applications of nanotechnological innovations in the development of advanced treatment modalities for individuals with PD.

## 1 Introduction

Parkinson’s disease (PD) is the second most common neurodegenerative disorder and affects 7–10 million people worldwide. The incidence rate of PD is 41 per 100,000 individuals, increasing to 1,900 per 100,000 individuals older than 80 years. It is projected that by 2040, the incidence rate will reach 12.9 million cases [1]. PD is a debilitating disease that significantly reduces patients’ quality of life, increases medical costs, and poses a social burden. PD can be characterized by motor and nonmotor symptoms. Motor symptoms include movement disorders, postural instability, and tremors [2], while nonmotor symptoms encompass sleep disorders, depression, anxiety, and loss of smell [3]. A pathological hallmark of PD is the progressive loss of dopaminergic neurons in the substantia nigra pars compacta (SNpC), which leads to a reduction in dopamine (DA) levels in the brain; this neurotransmitter plays a vital role in regulating movement, emotion, and cognitive function [4]. Another characteristic feature of PD is the presence of Lewy bodies (LBs), which are intracellular inclusions consisting of abnormal α-syn [5]. Although α-syn typically participates in neuronal signal transmission, its abnormal aggregation in PD leads to impaired neuronal function.

The exact etiology of PD has not been determined, but it is believed to be influenced by both genetic and environmental factors [6]. Approximately 5–10% of PD patients have a family history associated with certain gene mutations (LRRK2, PARK7, PINK1, Parkin RBR E3 Ubiquitin Protein Ligase [PRKN], and SNCA), while the majority of cases (90–95%) are sporadic and potentially linked to environmental toxin exposure (pesticides, heavy metals, organic solvents). Sex also plays a role in PD susceptibility, with men being more prone to PD than women, particularly before menopause, due to the neuroprotective effects of estrogen [7,8]. Both genetic factors in familial PD and environmental factors in sporadic PD affect specific pathways, such as mitochondrial dysfunction, oxidative stress, neuroinflammation, and abnormal protein degradation pathways, contributing to the clinical manifestations of PD [9,10]. Currently, there is no cure for PD, and treatment primarily focuses on symptom relief. Drugs such as levodopa, DA agonists, monoamine oxidase B (MAO-B) inhibitors, and catechol-*O*-methyltransferase inhibitors are used to restore DA levels and alleviate associated symptoms [11,12,13]. However, these drugs are limited by their ability to cross the blood‒brain barrier (BBB), susceptibility to peripheral metabolism, drug resistance, and side effects. Surgical treatments, such as deep brain stimulation (DBS), focused ultrasound (FUS), and cell replacement therapy [14], are options when drug therapy fails, but they provide only temporary improvement in motor symptoms without preventing neuronal death.

To overcome the limitations of PD treatment, novel strategies are needed. Nanotechnology has emerged as a promising approach in recent years, offering improvements in drug bioavailability/stability, overcoming biological barriers (including the BBB), reducing side effects, targeting disease sites precisely, and enabling real-time tracking. Nanotechnology can revitalize potentially valuable PD treatments with limited efficacy. This review provides an overview of PD mechanisms, current treatments, and ongoing clinical trials. The study also explores the challenges associated with delivering PD drugs to the central nervous system (CNS) and discusses how nanotechnology can address these obstacles. Furthermore, the review highlights the latest advancements in utilizing nanotechnology-based strategies to target specific pathophysiological aspects related to PD progression.

## 2 Proposed mechanisms of PD

Although the exact mechanisms of PD are not fully understood, they are believed to be mediated by complex interactions between cells, molecules, and genetic pathways. The currently proposed main mechanisms of PD include the following: (1) Loss of DA neurons: the primary feature of PD is the loss of dopaminergic neurons, which are responsible for motor regulation. The loss of dopaminergic neurons leads to a decrease in DA levels, resulting in symptoms such as motor disorders. (2) Protein misfolding and aggregation: Abnormal aggregation of α-syn occurs inside DA neurons, forming inclusions called LBs. These aggregates may cause neuronal dysfunction and cell death. (3) Oxidative stress: An imbalance between oxidants and antioxidants in the body leads to excess production of reactive oxygen species (ROS) or reactive nitrogen species (RNS) or insufficient clearance, resulting in cell damage. PD patients exhibit significant oxidative stress responses in the brain, manifested by increased lipid peroxidation, protein carbonylation, DNA damage, etc. Oxidative stress may promote PD development through various pathways, including increasing α-syn misfolding and aggregation, impairing mitochondrial function and structure, and activating neuroinflammatory responses. (4) Mitochondrial dysfunction: Mitochondrial dysfunction related to energy metabolism, cell apoptosis, calcium ion balance, etc. leads to insufficient cellular energy and oxidative stress. PD patients exhibit significant mitochondrial dysfunction in the brain, manifested as decreased mitochondrial respiratory chain enzyme activity, mitochondrial DNA (mtDNA) mutations or deletions, reduced mitochondrial membrane potential, and impaired mitochondrial autophagy. Mitochondrial dysfunction may affect PD pathogenesis through various pathways, including increasing α-syn misfolding and aggregation, releasing cytochrome c and proapoptotic factors, and inducing neuroinflammatory responses. (5) Neuroinflammation: Sustained or excessive inflammatory responses in the nervous system lead to neuronal damage or death. PD patients exhibit significant neuroinflammation, manifested by the activation of glial cells (such as astrocytes and microglia), the release of proinflammatory cytokines (such as TNF-α, IL-1β, and IL-6), and the infiltration of immune cells (such as T cells and B cells). Neuroinflammation may exacerbate PD progression through various mechanisms, including inducing mitochondrial dysfunction, increasing the production of ROS or RNS, and promoting α-syn transfer and diffusion. (6) Genetic factors: Some PD cases are associated with genetic mutations, such as LRRK2, PINK1, Parkin and Wnt/β-catenin (WβC) [15] gene mutations. These mutations may affect mitochondrial function, α-syn metabolism, and other pathways related to PD. Currently, treatment strategies targeting these mechanisms have made some progress in PD clinical trials and research. In Section 3, a brief introduction of approved PD treatments and ongoing clinical trials is given.

## 3 Current treatments and clinical trials for PD

Table 1 shows the various therapeutic drugs employed in the management of PD, with a primary focus on reinstating dopaminergic neurotransmission in neurons. Additional drugs targeting the glutamatergic, noradrenergic, serotonergic, and cholinergic systems are also utilized. Directly supplementing exogenous DA to counteract the depletion caused by the loss of dopaminergic neurons is the most effective approach. However, the hydrophilicity of DA poses challenges in crossing the BBB, and its short half-life and potential long-term adverse reactions limit its use. Hence, the current practice involves administering levodopa, a DA precursor that is converted into DA in the brain, which has therapeutic effects.

**Table 1:** Ongoing phase III interventional trials on PD

Compound	Target/mechanism	Recruiting	Identifier
Apomorphine	DA receptor agonist	Not yet	NCT02339064
Exenatide	Neuroprotective	Not yet	NCT04232969
Apomorphine	DA receptor agonist	Yes	NCT02864004
Carbidopa and levodopa	Increase DA levels	Not yet	NCT04006210
Pimavanserin tartrate	Modulating the activity of 5-HT2A receptors	Yes	NCT06068465
ABBV-951	Supplement DA and inhibit DA degradation	Not yet	NCT04750226
ABBV-951	Supplement DA and inhibit DA degradation	Not yet	NCT04379050
Buntanetap	Anti-inflammation, neuroprotective, regulation of protein metabolism	Not yet	NCT05357989
Tavapadon	Partial agonist of DA D1/D5 receptors	Enrolling by invitation	NCT04760769
Apomorphine	DA receptor agonist	Yes	NCT04879134
Tavapadon	Partial agonist of DA D1/D5 receptors	Yes	NCT04223193
Tavapadon	Partial agonist of DA	Yes	NCT04201093
Conventional medication/Chinese herbal medicine	Neuroprotective	Yes	NCT05001217
Botulinum toxin type A	Neuromuscular blocker	Yes	NCT04277247
Rasagiline	MAO-B inhibitor	Not yet	NCT05611372
*Lactobacillus acidophilus*	Anti-oxidative effect, immune modulation, regulation of gut microbiota, and modulation of neurotransmitters	Yes	NCT05576818
Tavapadon	Partial agonist of DA D1/D5 receptors	Not yet	NCT04542499
Dipraglurant	Modulating the glutamatergic neurotransmitter system	Yes	NCT04857359
Dipraglurant	Modulating the glutamatergic neurotransmitter system	Yes	NCT05116813
Opicapone	Catechol-*O*-methyltransferase (COMT) inhibitor	Not yet	NCT04978597
Nicergoline	Vasodilation, activation of α-adrenergic receptors, and improvement of cerebral metabolism	Not yet	NCT05551182

DA receptor agonists such as ropinirole (Requip), pramipexole (Mirapex), piribedil (Trivastal), and rotigotine (Neupro) directly stimulate DA receptors, improving both motor and nonmotor functions [16]. MAO-B inhibitors such as selegiline, rasagiline, and safinamide effectively prevent DA level reductions and the production of toxic intermediates by inhibiting MAO-B. These inhibitors can be used alone or in combination with other medications to prolong levodopa’s action and reduce “off” periods in early- or mid-to-late-stage patients. COMT inhibitors such as entacapone (Comtan) and tolcapone (Tasmar) inhibit the peripheral metabolism of levodopa, increasing its concentration in the brain [17]. These agents are combined with levodopa for mid-to-late-stage patients who experience motor fluctuations, particularly “wearing-off” phenomena, to extend the duration of action and reduce the dosage.

Anticholinergic drugs such as trihexyphenidyl and benztropine rebalance DA and acetylcholine by inhibiting cholinergic neuron activity [18]. In PD, elevated levels of glutamate, a neurotransmitter, lead to neuronal loss and disease progression. Along with associated complications, anti-glutamatergic drugs such as amantadine (a glutamate/N-methyl-D-aspartic acid [NMDA] receptor antagonist) partially alleviate motor and nonmotor symptoms in PD patients.

Surgical treatment, which is primarily suitable for mid-to-late-stage patients who have an inadequate response to medications or severe complications, serves as an alternative therapeutic approach for PD. DBS, involving the implantation of microelectrodes into the patient’s brain, is a widely used surgical technique. It utilizes high-frequency electrical stimulation to suppress abnormal neural activity and improve motor impairments. DBS preserves brain tissue, resulting in minimal complications while enabling adjustments to stimulation parameters for long-term therapeutic effects [14]. Internationally, DBS has been recognized as the gold standard for surgical treatment of mid- to late-stage PD.

Research efforts to develop additional drugs and treatment strategies for PD through clinical trials have been ongoing in recent years. These include drugs targeting gene mutations, such as LRRK2, PINK1, and Parkin; excitotoxicity inhibitors; mitochondrial protectants; antiapoptotic agents; anti-inflammatory drugs; and neurotrophic factors (NTFs). Table 1 provides a list of ongoing phase III intervention trials based on data from the United States National Library of Medicine. The future holds promise for an increasing number of available drugs for PD treatment. However, considering the complex nature of PD etiology, drugs targeting individual mechanisms may not yield significant therapeutic effects. Hence, combination therapy may improve clinical outcomes. Furthermore, challenges persist in effectively delivering therapeutic drugs to the CNS to slow the clinical progression of PD due to biological barriers (e.g., the BBB and blood–spinal cord barrier [BSCB]) and inherent limitations of drugs (e.g., low solubility, susceptibility to degradation, and off-target effects). Section 4 provides a detailed discussion of the challenges involved in delivering therapeutic drugs to the CNS for PD treatment.

## 4 Challenges in delivering therapeutic PD drugs to the CNS

### 4.1 BBB

**Figure 1:**
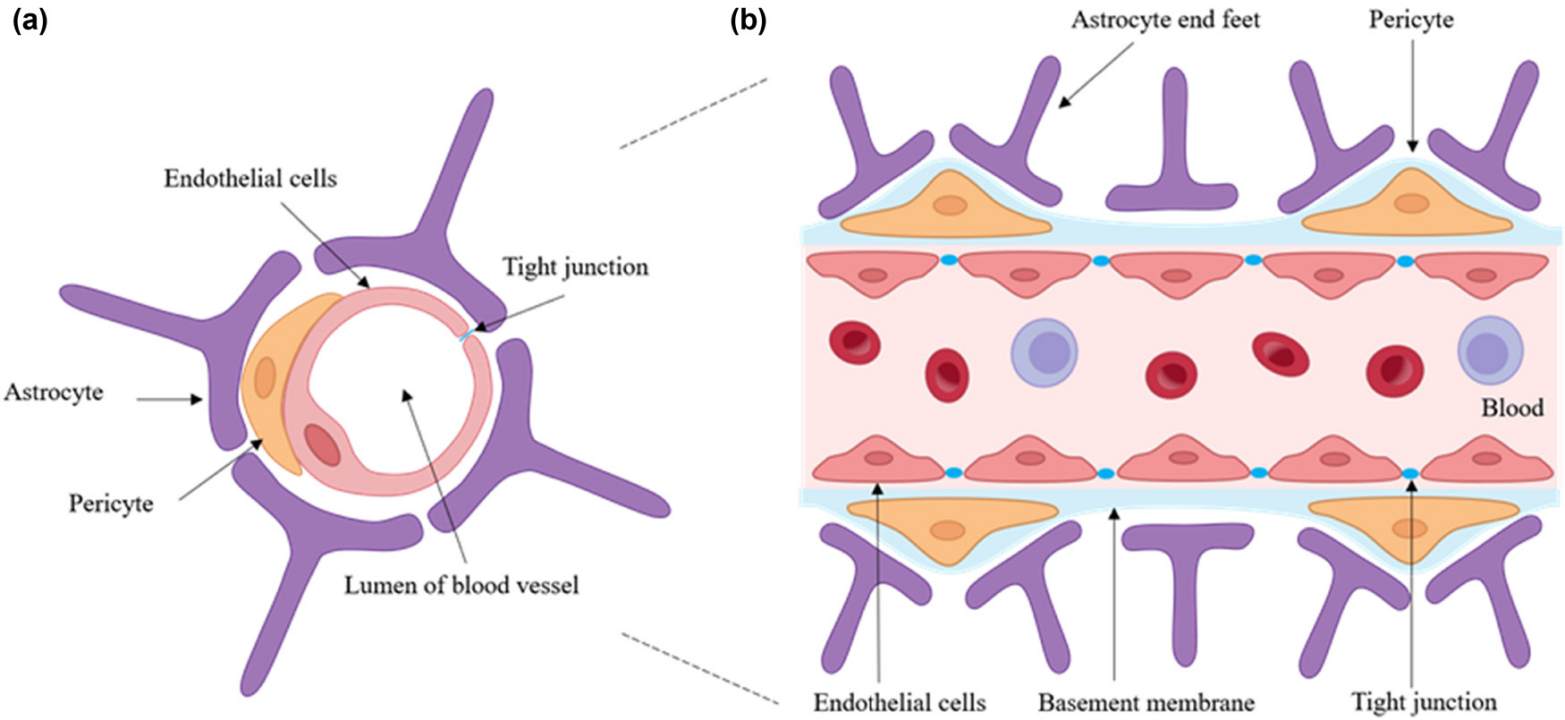
Schematic illustration of the BBB. General structure of the BBB. Figure 1a and b shows different cross-sectional views of brain blood vessels, respectively. The BBB is formed by ECs with tight junctions, surrounded by pericytes and astrocyte end feet. The image shows the direction of cross-section of (a) (cut in round slices) and (b) (cut into squares) when the brain blood vessels are viewed as a cylinder.

Several anatomical and metabolic barriers, including the cerebrospinal fluid (CSF), the choroid plexus, and the BBB, exist between the peripheral blood circulation and the brain. The BBB is one of the most important and highly selective barriers that regulates the movement of various substances between the bloodstream and the CNS, thereby playing a crucial role in maintaining brain homeostasis. The BBB is composed of endothelial cells (ECs), vascular smooth muscle cells, neurons, astrocytes, and perivascular macrophages (Figure 1) [19]. The ability of the BBB to act as a selectively permeable filter is achieved through the presence of tight junctions and adherens junctions in the endothelial lining of the BBB. The tight junctions seal the gaps between ECs, creating a continuous lumen within the blood vessels. On the other hand, adherens junctions facilitate cell‒cell contact among ECs [20,21].

The molecular mechanisms of the transport of substances across the BBB include efflux and influx mechanisms. Transcellular pathways are crucial for transporting lipophilic agents, whereas paracellular pathways are responsible for transporting hydrophilic molecules [22]. Transport across the BBB occurs through endogenous carrier-mediated transport (CMT) or a receptor-mediated transport (RMT) system [23]; through adsorptive-mediated transcytosis (AMT); or through active efflux, facilitated by various major transporters, receptors, and channels present in ECs and pericytes. CMT is accountable for transporting vitamins, hormones, organic anions/cations, and nucleotides across the BBB. This process entails the spontaneous passive transport of these molecules through transmembrane integral proteins along a concentration gradient. The RMT pathway is the primary pathway for transporting essential macromolecules for brain function across the BBB. The ECs that form the BBB are equipped with receptors that specifically recognize certain ligands. When these receptors engage with their corresponding ligands, a process is triggered within the cell membrane that causes them to fold inwards, creating specialized intracellular compartments called coated vesicles. These vesicles undergo intricate processing mechanisms, allowing for the efficient recycling of both the receptor and the ligand. Ultimately, recycled receptor‒ligand complexes are released at the basolateral surface of ECs, where they contribute to the dynamic regulation of BBB function [24]. Large molecules such as antibodies, transferrin, insulin, and low-density lipoprotein (LDL) are transported through RMT. This process involves specific transporters, including insulin receptor, insulin-like growth factor receptor, type 1 transferrin receptor, low-density lipoprotein receptor (LDLR), leptin receptor, and neonatal Fc receptor.

The concept of AMT emerged from the observation that polycationic peptides not only have the ability to bind to the surface of ECs but also possess the remarkable capability to penetrate the BBB [25]. AMT involves the interaction of positively charged (cationic) and negatively charged (anionic) molecules on the EC membrane, facilitating the transport of positively charged proteins and basic short peptides, such as cell-penetrating peptides.

Efflux pumps, including P-glycoprotein (P-gP), ATP-binding cassette transporters (ABCs), and multidrug resistance proteins, establish a formidable barrier that hinders the passage of drugs across the BBB. P-gP, identified as ATP-binding cassette subfamily B member 1 (ABCB1), is a prominent ABC efflux transporter located on the luminal membrane of brain capillary ECs [26]. In cancer cells, the active efflux of drugs by P-gP contributes to the emergence of multidrug resistance, whereby a wide range of drugs, such as paclitaxel, digoxin, and dexamethasone, can function as substrates for this transporter and are actively expelled into the bloodstream [27]. Administration of paclitaxel in combination with a potent P-gP inhibitor in a mouse model resulted in a notable increase in the brain accumulation of paclitaxel [28].

Although numerous drugs are utilized for the treatment of neurological disorders, the specialized microvasculature of the BBB limits the use of only a few medications during systemic administration, leading to inadequate efficacy. Research endeavors have been directed toward the advancement of innovative approaches to transport drugs to the CNS. These approaches include the utilization of in-deliver drug-conjugated nanoparticles (NPs), liposomes, albumin NPs, and solid lipid nanoparticles (SLNs). By leveraging the intrinsic transport systems within the ECs of cerebral capillaries, small pharmaceutical drugs capable of traversing the BBB can be designed.

### 4.2 Biostability and bioavailability

In addition to the selective restrictions imposed by the BBB, the efficacy of potential therapeutic agents for PD faces significant challenges due to inadequate bioavailability and biostability of the drugs. Notably, many neuroprotective agents developed by the pharmaceutical industry have problems associated with low aqueous solubility. For instance, coenzyme Q10, an antioxidant, exhibits poor solubility in water, which hampers its therapeutic potential in PD treatment [29]. Similarly, a compound known as a Nurr1 agonist has shown promise in combating PD in animal models but is also limited by poor water solubility, necessitating improvements in pharmacological formulations to enhance its effectiveness [30]. In clinical practice, the majority of drugs employed for treating CNS disorders are small molecules that typically possess high lipophilicity and can traverse the BBB via active or passive mechanisms. However, drug molecules that are too lipophilic might accumulate in the ECs of brain vessels, preventing effective delivery to the deep brain regions where DA neurons – targeted in PD – are located. Therefore, designing drug molecules with a proper balance of lipophilicity and aqueous solubility is essential for ensuring successful accumulation and distribution of these drugs within the CNS for PD treatment. Moreover, emerging biologic therapies, such as gene-editing tools, NTF therapy, and antibody therapy, have demonstrated potential in PD treatment. For example, the use of adeno-associated virus vectors to deliver specific genes can restore the function of impaired DA neurons in PD models [30,31]. However, these large biomolecular drugs typically struggle to cross the BBB and are susceptible to degradation by enzymes in the body, which limits their clinical utility. To surmount these barriers, the application of nanotechnology provides a promising approach. Encapsulating drug molecules in nanocarriers can improve their stability in the bloodstream, enhance their capacity to cross the BBB, and facilitate targeted release in specific brain regions. This methodology has the potential to increase the bioavailability of PD therapeutics, thereby offering more effective treatment modalities for patients with PD.

### 4.3 Systemic distribution and clearance

Due to the lipophilic properties of many drugs, they exhibit extensive distribution in the body, potentially compromising their specificity. This may necessitate an increase in drug dosage to achieve the desired therapeutic concentration. However, this nonspecific systemic distribution can lead to heightened systemic toxicity and adverse effects on patient quality of life. For instance, upon oral administration, levodopa requires absorption through the intestine into the bloodstream and entry into the brain via the amino acid transport system [32]. However, it also undergoes widespread distribution in other bodily tissues, resulting in a spectrum of side effects, including cardiovascular and gastrointestinal discomfort. Additionally, the rapid systemic clearance of numerous anti-Parkinson’s drugs significantly impacts the concentration of drugs that reach the brain. For instance, l-3,4-dihydroxyphenylalanine (l-DOPA) may undergo enzymatic and nonenzymatic degradation in the gastrointestinal tract after oral administration. While combining carbidopa or benserazide can reduce levodopa metabolism in the body, the primary issue lies in the rapid clearance of hydrophilic drug molecules by the kidneys due to poor reabsorption, whereas lipophilic drugs are typically converted into hydrophilic metabolites in the liver before excretion via the kidneys or bile. Consequently, the therapeutic efficacy of drugs for PD may be influenced by their systemic distribution and rapid systemic clearance.

## 5 Lipid-based NPs for PD

### 5.1 Classification of lipid-based NPs

**Figure 2:**
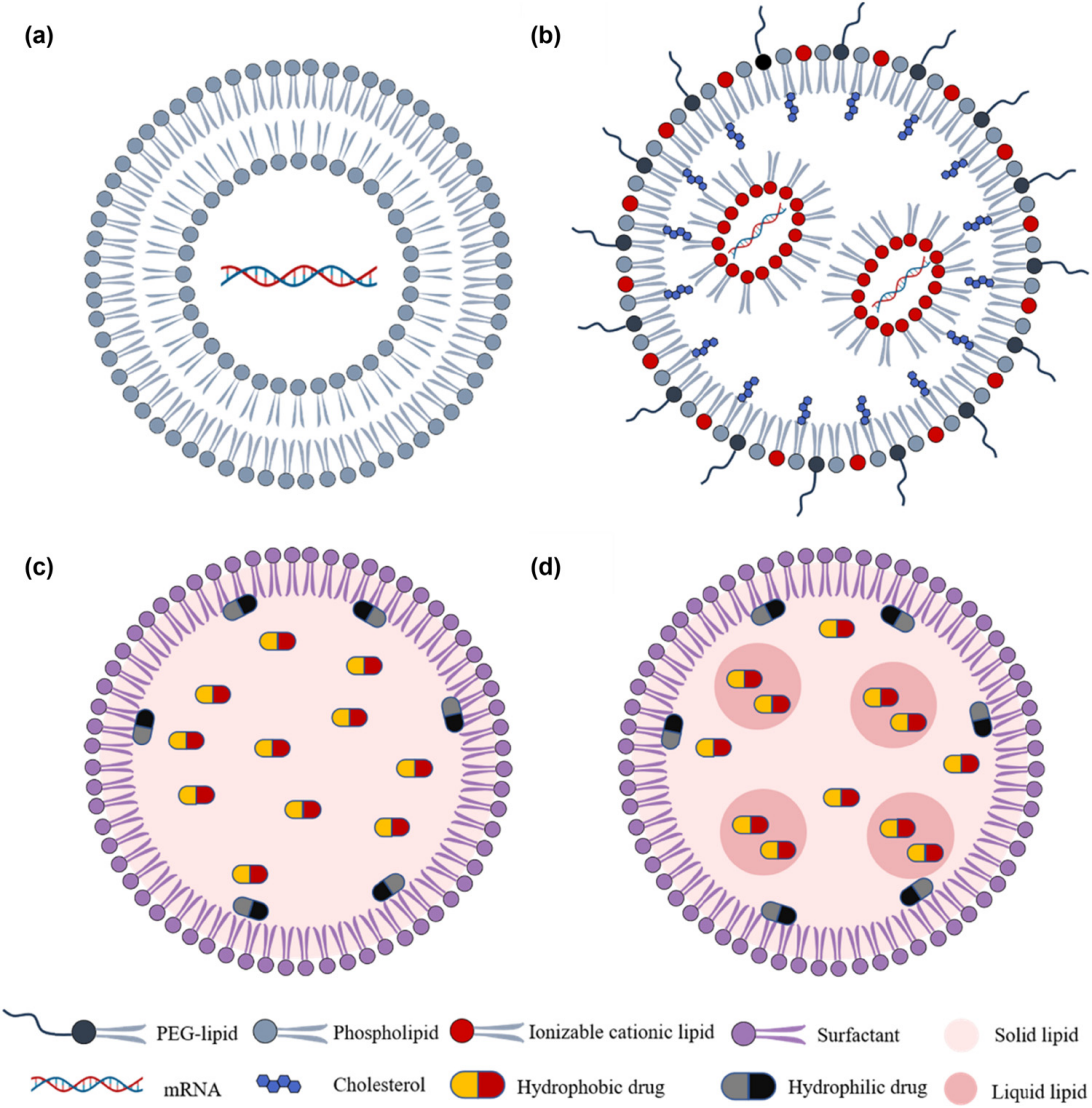
Schematic representation of lipid-based NPs. They are classified according to the physical state of their lipidic component in NPs, which include (a) liposomes, (b) LNPs, (c) SLNs, and (d) NLCs.

Lipid-based nanocarriers are commonly preferred over other types of nanocarriers due to their ability to mimic the natural lipid environment of biomembranes and their lower toxicity when administered *in vivo* [33]. Additionally, they can effectively utilize physiological transport mechanisms, such as the LDL receptor pathway, to facilitate delivery to the brain. Lipid-based NPs can be classified into several main types, including liposomes, lipid nanoparticles (LNPs), SLNs, and second-generation LNPs known as nanostructured lipid carriers (NLCs) (Figure 2).

#### 5.1.1 Liposomes

Liposomes, as the first generation of colloidal nanocarriers, have been widely recognized as effective drug delivery systems (DDSs) since the 1970s [34]. Owing to the hydrophilic core enveloped by one or multiple phospholipid bilayers resembling cell membranes, liposomes are strategically utilized for the targeted delivery of drugs, proteins, and peptides [35]. Based on their size and number of bilayers, liposomes can be categorized into three types: small unilamellar (10–50 nm), large unilamellar (50–1,000 nm), and multilamellar (20–100 nm) liposomes. These reversible structures are amenable to noncovalent interactions, such as van der Waals forces and hydrogen bonding among molecules [36].

The phospholipid bilayer of liposomes holds promise for transporting medications across the BBB. However, BBB crossing is strictly regulated. To enhance liposomal carrier transport through the BBB, various surface modifications have been investigated [37]. The BBB surface contains diverse proteins, peptides, antibodies, and ligand receptors. Surface-active ligands derived from these compounds show potential for facilitating transcytosis and cationic liposome absorption into the BBB. Nutrient coatings, such as glucose, are commonly applied to liposomes for optimal systemic distribution. Upon successful BBB traversal, passive diffusion mechanisms driven by brain efflux occur [38].

#### 5.1.2 LNPs

Lipid nanoparticles (LNPs) are an advanced nanoscale drug delivery system, typically with sizes controlled between 20 and 200 nanometers, which facilitates their circulation within biological systems and penetration of biological barriers. LNPs are spherical vesicles composed of cationic or ionizable lipids, cholesterol, phospholipids, and polyethylene glycol (PEG) lipids, capable of stably encapsulating and delivering drug molecules. These nanoparticles possess low cytotoxicity, exceptional drug encapsulation capacity, high biocompatibility, and minimal immunogenicity potential, making them optimal candidates for targeted therapies in the treatment of central nervous system (CNS) diseases [39]. Below is a detailed introduction to the main components of LNPs.

Initially, cationic lipids were used because they not only bind anionic RNA but also fuse with the membrane to promote cellular uptake and endosomal escape. Although cationic lipids have shown promising efficacy for successful delivery, the permanent charge they carry results in high cytotoxicity and limits their potential applications. Ionizable lipids have been used instead of permanent cationic lipids to avoid hemolysis and to enable rapid clearance from the circulation because of their positive charge. Ionizable lipids can change charge based on their environment, portraying a neutral charge at physiological pH with low toxicity and a positive charge at low pH that permits nucleic acid complexation. Several parameters, such as tail length, unsaturation, branching, and pKa, significantly influence the properties of ionizable lipids. The unsaturation of ionizable lipids affects their pKa and fusion capability [40]. Reduced unsaturation increases pKa values, facilitating the ionization of ionizable lipids in alkaline environments. Appropriate unsaturation enhances fusion with cell membranes, promoting efficient gene or drug delivery. Similarly, longer lipid tails increase hydrophilicity and solubility, improving stability and manipulability *in vitro* and *in vivo*. An appropriate tail length allows for effective targeting and delivery to specific cells or tissues.

Phospholipids and cholesterol serve as helper lipids to enhance cellular internalization, facilitate membrane fusion, and promote escape from the endosome [41]. Specifically, cholesterol fills interstitial gaps between lipids in the liposomal membrane, thereby stabilizing the LNP structure.

PEGylation, the covalent attachment of PEG to lipids, is a widely used strategy to prolong the circulation half-life of lipids [42]. This approach effectively reduces macrophage-mediated clearance and inhibits apolipoprotein adhesion [43]. Additionally, PEG modification enhances steric stability, thereby extending the storage time of lipid-based formulations [44].

#### 5.1.3 SLNs

SLNs are lipid-based biocompatible nanocarrier systems that primarily consist of lipids or modified lipid derivatives (e.g., triglycerides, fatty acids, and waxes) that exhibit nanostructures ranging in diameter from 10 to 1,000 nm. SLNs have a solid hydrophobic lipid core in which both hydrophilic and lipophilic drugs can be dispersed [45]. The physicochemical properties of SLNs, such as size, polydispersity, surface charge, stability, drug loading, and release profile, are greatly influenced by the careful selection of lipids, surfactants, and formulation composition. SLNs have demonstrated their remarkable ability to effectively transport both lipophilic and hydrophilic drugs to various diseased tissues. Moreover, they offer a versatile delivery system for not only drugs but also therapeutic molecules such as oligonucleotides, peptides, genes, and even smaller NPs such as superparamagnetic iron oxide NPs. This versatility positions SLNs as a highly promising platform for targeted drug delivery in various biomedical applications.

There are various patterns of SLN formulations according to the distribution of the drug components within them. According to the Drug-Enriched Shell Model, the core of the SLN is drug free, and the main active drug is distributed around the outer shell of the SLN. A hot homogenization process is utilized, causing the lipid content to precipitate at the core while leaving the drugs at the outer shell upon cooling. This model enables faster drug release due to the large surface area of the outer layer, with burst release controlled through larger drugs or adjusted surfactant formulations [46,47]. According to the drug-enriched core model, the active drug is concentrated within the SLN and is encased by the lipid outer shell. The drug is first liquified in the lipid, leading to supersaturation, and then concentrated at the center upon cooling. Drug release is controlled by the nature of the lipid membrane and follows Fick’s law of diffusion [48]. According to the homogeneous matrix model, the active drug is distributed within the lipid matrix of the SLN. The drug may exist in dispersion or in amorphous clusters within the lipid. Lipophilic drugs can be encapsulated in the lipid matrix without the use of surfactants. The drug release profile is extended in this model due to the strong molecular dispersion of drug particles in the colloidal matrix [46].

SLNs possess high biocompatibility and reduced systemic toxicity due to their physiological solid lipid emulsion system and minimal organic solvent usage [49]. This, in turn, allows for controlled and sustained drug release through their solid lipid composition, resulting in extended diffusion pathways and enhanced therapeutic efficacy [50]. Moreover, the lipid matrix of SLNs offers effective protection against biochemical degradation, ensuring improved drug stability [51]. With their ability to be tailored for active targeting of specific tissues and crossing the BBB, SLNs facilitate drug accumulation at desired sites of action [52]. Extensive studies have demonstrated that SLNs not only improve the pharmacokinetics, tissue distribution, and bioavailability but also lead to improved therapeutic outcomes. The scalability of SLN production utilizing easily obtainable raw materials enables cost-effective large-scale industrial manufacturing [53]. Additionally, the affordability of the sterilization and lyophilization processes further supports the wide application of SLNs. Furthermore, SLNs exhibit versatility in delivering a diverse range of drugs, including both hydrophilic and lipophilic compounds, while also accommodating traditional pharmaceutical formulations and biomolecules.

Although SLNs have many advantages, they also have several disadvantages, such as therapeutic expulsion, gelation tendency, and low encapsulation efficiency (EE) [54]. The low EE is primarily due to the internal structure of the lipid core, which lacks empty spaces during crystallization, making it challenging for the encapsulated substance to remain within the solid phase. To address this issue and enhance the loading efficiency of SLNs, Müller et al. [55] proposed modified SLNs with structural defects in the solid lipid core, increasing the internal free space for larger payloads. This advanced generation of SLNs not only improved the loading efficiency but also exhibited enhanced stability and prevented drug expulsion during storage [56].

#### 5.1.4 NLCs

In the late 1990s, as the second generation of lipid nanocarriers, NLCs were developed to overcome the limitations associated with SLNs. These limitations include poor drug-loading capacity due to the highly organized crystalline structure and the tendency toward drug expulsion during storage caused by lipid crystallization [57]. In contrast, NLCs consist of a combination of solid and liquid lipids at a 70:30 to 99.9:0.1 ratio, along with an aqueous phase consisting of a surfactant [58]. This composition allows for the formation of a less ordered crystalline structure, which provides additional space for drug loading. Moreover, it reduces the crystallinity of the lipid matrix and prevents drug expulsion [59].

NLCs can be classified into three types based on their preparation methods, lipid matrix structure, and drug location [59]. The first type is the imperfect NLCs, which result from blending different lipids and yield a disorganized lipid matrix. This type of oil typically consists of a small amount of liquid oil mixed with a larger quantity of solid lipids. The lipids used may vary in terms of fatty acid origin, carbon chain length, or degree of saturation. Altering the type of fatty acid triglyceride used allows for modifications in the NP structure and imperfections. Increasing the lipid concentration enhances drug incorporation, as lipophilic drugs have greater solubility in liquid lipids. The second type is amorphous NLCs, which incorporate specific lipids such as hydroxypropyl behenate and isopropyl myristate through a specific blending method of solid and liquid lipids. These lipids facilitate the formation of a noncrystalline matrix, preventing crystallization of the solid lipid core and minimizing drug leakage [60]. The third type is multiple-type NLCs, which consist of a solid lipid matrix and liquid oil. These NLCs can be further subdivided into two subtypes: oil-in-solid, lipid-in-water type; and oil-in-water, lipid-in-water type. In oil-in-solid, lipid-in-water-type NLCs, numerous nanocompartments of liquid oil are embedded within a solid lipid matrix. This unique structure enhances drug bioavailability and enables controlled drug release [61]. On the other hand, oil-in-water, lipid-in-water-type NLCs comprise multiple oil droplets surrounded by lipids and dispersed in an aqueous phase. They exhibit a similar mechanism of drug release to oil-in-solid, lipid-in-water-type NLCs.

NLCs are influenced by various factors, shaping their properties. The lipid composition is crucial because different components affect the stability, drug entrapment efficiency, and release performance of a material. A high content of saturated fatty acids enhances physical stability, while unsaturated fatty acids or solid oils improve drug entrapment. Surfactant selection impacts the stability, particle size, and drug release rate, with hydrophilic and lipophilic properties influencing the interfacial properties. Processing methods, such as high-pressure homogenization, ultrasonic emulsification, and solvent precipitation, affect the microstructure, drug entrapment, and dispersibility. Environmental factors, including temperature, humidity, and pH, influence stability and drug release.

### 5.2 Designing advanced therapeutic lipid-based NPs specifically targeting PD pathophysiology

Overcoming the hurdle of effectively delivering therapeutic drugs, trophic factors, and biomacromolecules through the BBB and BSCB to access the CNS remains a formidable challenge in treating PD. However, there is promise in the rapid development of nanotechnologies, which offer potential solutions to the multifaceted barriers currently impeding the effectiveness of PD treatments, as previously discussed. This section revisits the various mechanisms underlying PD pathophysiology and delves into the detailed exploration of the design and utilization of nanomaterials to address these challenges (Table 2). These discussions provide an impetus to transform nanomedicine from a theoretical concept into practical clinical trials for PD treatment.

**Table 2:** LNP-based drug delivery for PD

Biological target	Nanocarrier	Encapsulated drug/genes	Reference	Biological target	Nanocarrier	Encapsulate drug/genes	Reference
DA replacement	Rabies virus glycoprotein (RVG29)-liposomes	*N* 3, 4bis(pivaloyloxy)-DA	[63]		Liposome	Resveratrol (RES)	[95]
	CITX-Liposomes	l-DOPA	[64]		Chitosan-SLNs	Curcumin	[158]
	Transferrin-liposomes	DA	[131]		SLN	Trehalose	[159]
	Chitosan-liposomes	l-DOPA	[65]		SLN	Quercetin	[160]
	Amyloid precursor protein (APP)-liposome	DA	[66]		SLN	Curcumin	[161]
	Liposomes	L-DOPA	[134]		SLN	GER-UDCA	[162]
	Liposomes	DOPH	[135]		Polysorbate 80-SLN	Formoterol	[100]
	OX26 monoclonal antibody (Mab)-PEG-Liposomes	DA	[136]		SLN	*Bacopa monnieri*	[163]
	Maltodextrin-Liposomes	Levodopa	[137]		SLN	Citicoline	[164]
	Fe_3_O_4_-NMD-Liposomes	Nimodipine	[138]		Nanoemulsion	Vitamin E and RES	[91]
	Liposomes	DA	[139]		Lipid- poly(lactic-co-glycolic acid) (PLGA) bubbles	Curcumin	[97]
	Liposomes	L-DOPA prodrugs	[140]		LNPs	Curcumin and piperine	[165]
	SLN	L-DOPA	[141]		PUFA-PL-LCLNs	NPs themselves served therapeutic purpose	[166]
	SLN	Ropinirole hydrochloride	[142]	Neurotrophic factor	Liposome	Glial cell line-derived neurotrophic factor (GDNF)	[108]
	SLN	Pyrrolobenzodiazepine	[143]		Liposome	Recombinant human fibroblast growth factor-20	[111]
	SLN	Bromocriptine mesylate	[68]		Liposome	GDNF	[93]
	SLN	Apomorphine	[70]		TAT-NLC	GDNF	[95]
	SLN	Piribedil	[144]		NLC	Basic fibroblast growth factor (bFGF)	[110]
	SLN	DA	[145]		Chitosan-NLC	GDNF	[119]
	SLN/liposomes	DA	[146]		Lipid nanomicro-bubble	GDNF	[120]
	NLC	Apomorphine diester prodrugs	[147]		Lipid nanomicro-bubble	Nrf2	[167]
	NLC	L-DOPA codrugs	[148]		RVG-liposomes	α-syn small interfering RNA (siRNA)	[114]
	NLC	Bromocriptine	[69]	Gene therapy	Mab-liposome	GDNF plasmid	[107]
	SLN and NLC	Ropinirole	[67]		Liposome	GDNF plasmid	[120,121]
	LNP	Apomorphine	[149]		PEG-immunoliposomes	Tyrosine hydroxylase (TH) plasmid	[116,168,169]
	LNP	L-DOPA precursor	[150]		LNP	Csf2 mRNA	[117]
	SLN	DA	[151]		LNP	GRIN1 siRNA	[115]
	Lipid-polymer hybrid NP	L-dopa	[152]		LNP	L-PGDS ASO	[118]
	Lipid-polymer hybrid NP	Ropinirole hydrochloride	[153]		LNP	Albumin-GM-CSF mRNA	[170]
	SLN	Bromocriptine and RES	[154]		SLN	NPs themselves served therapeutic purpose	[128]
	Polysorbate80-liposomes	Curcumin	[79]	Stem cell therapy	Heparinized cationic SLN	NGF-hcsln	[129]
Alpha-Synuclein Aggregates	Leptin (Lep)-liposome	RES and epigallocatechin gallate (EGCG)	[78]		Ln5-P4-ASLNs	NGF	[78]
	Mab-Liposomes	SynO4	[80]		Lipid-based NPs	miRNA	[131]
	PEG-cholesterol nanoliposomes (NLPs)	Baicalein	[155]		LNPs	mRNA	[132]
	Chol-PEG-Zwitterionic NLPs	NPs themselves served therapeutic purpose	[81]				
	Antitransferrin receptor antibody-liposomes	pomace seed extract	[156]				
Oxidative stress, mitochondrial dysfunction and inflammation	Fe_3_O_4_-liposome	RES	[157]				
						
						

#### 5.2.1 DA replacement

DA is synthesized from tyrosine through the action of an enzyme called TH, which converts tyrosine into l-DOPA. l-DOPA is then decarboxylated by aromatic l-amino acid decarboxylase to form DA. Once synthesized, DA is stored and protected within small synaptic vesicles by vesicular monoamine transporter 2 until it is ready for release into the synaptic cleft. This storage mechanism ensures the maintenance of low levels of free cytoplasmic DA, as DA is prone to oxidation and the generation of potentially neurotoxic substances under intracellular conditions. In the striatum, a brain region, DA exerts its effects by interacting with five distinct types of DA receptors. Notably, D1R and D2R (receptors 1 and 2, respectively) play crucial roles in the regulation of motor control signaling pathways. D1R facilitates movement via direct pathways, whereas D2R inhibits movement through indirect pathways. Upon DA binding to these receptors, a cascade of signal transduction pathways is initiated, modulating the excitatory or inhibitory state of neurons and leading to specific physiological effects.

After being released by dopaminergic neurons, DA quickly dissociates from its receptors and is taken back into dopaminergic neurons through the DA transporter (DAT) to clear the extracellular space of DA. DA, which is not reuptaken, is decomposed by enzymes called catechol-*O*-methyltransferases and monoamine oxidases in the presynaptic terminal. The breakdown process can generate hydrogen peroxide (H_2_O_2_), which can cause oxidative stress and neurotoxicity. After damage to the nigrostriatal pathway occurs, surviving dopaminergic neurons undergo adaptive changes to offset this loss. These changes involve heightened DA metabolism, elevated D2 DA receptor density, and diminished expression of DAT, all aimed at preserving appropriate levels of extracellular DA. These compensatory mechanisms serve to impede the progression of early-stage PD symptoms. Nevertheless, as the neurodegenerative process persists, DA depletion transpires, resulting in disruptions in motor control and the manifestation of symptomatic conditions.

Current therapies for PD focus on relieving motor symptoms by administering levodopa, a DA precursor or DA agonist (e.g., pramipexole, ropinirole, or rotigotine). Despite providing a temporary solution for controlling motor symptoms, the therapeutic efficacy of these drugs is constrained by various factors, including adverse effects, short half-lives in the systemic circulation and extracellular compartments, instability, poor bioavailability, and challenges in traversing the BBB. These shortcomings can be overcome through the delivery of drugs using different types of nanomaterials.

In the context of PD, liposomes loaded with DA have been engineered to facilitate prolonged and controlled release of this neurotransmitter. Klionsky et al. effectively encapsulated DA within liposomes and demonstrated its controlled release capabilities *in vivo*. The sustained release of DA from these liposomes resulted in increased DA levels within the striatum, leading to partial behavioral restoration [62]. With the confirmation of the feasibility of DA encapsulation and transport within liposomes, scientific investigations have shifted their attention toward surface modification to enhance the EE, targeting specificity, and controlled release in specific regions of the brain. For instance, DA-loaded RVG-29-conjugated liposomes effectively permeated the BBB when administered intravenously, leading to increased levels of DA- and TH-positive cells in the striatum [63]. ClTx-modified stealth liposomes loaded with levodopa showed promising therapeutic effects in a mouse model of PD, reducing behavioral disorders and protecting dopaminergic neurons, indicating their potential as a targeted DDS for improved PD therapy [64]. Cao et al. demonstrated that chitosan-coated levodopa liposomes could mitigate the induction of dyskinesias in PD patients. The mechanism may be associated with the signaling pathway involving phosphorylated ERK1/2, phosphorylated Thr34 DARPP-32, and ΔFosB within the striatum [65]. The surface modification of these liposomes with APP facilitates the penetration of DA through the BBB and leads to an increase in the striatal DA concentration [66].

Despite being nanosystems with great potential for controlled release, phospholipid-based liposomes have low stability and high costs, which has prompted the recent development of SLN and NLC. Dudhipala et al. developed SLNs and NLCs to encapsulate ropinirole. Pharmacokinetic and pharmacodynamic studies demonstrated a significant improvement in the oral and topical bioavailability of ropinirole, resulting in effective treatment of PD [67]. Similarly, Esposito et al. developed NLCs and SLNs as delivery systems for bromocriptine, an ergoline agonist of DA receptors. The encapsulation of bromocriptine in NLC/SLN can yield sustained therapeutic effects in a rat model of PD and prolong the half-life of bromocriptine *in vivo* [68,69]. The oral bioavailability and brain regional distribution of apomorphine, a DA receptor agonist used to treat PD, can be enhanced by utilizing SLNs as carriers, thereby improving the therapeutic efficacy of PD [70].

#### 5.2.2 α-Syn aggregation

α-Syn is a highly expressed neuronal protein in presynaptic terminals that, under normal physiological conditions, plays a role in regulating signal transmission and synaptic plasticity at the synaptic terminal. α-Syn is naturally unfolded in aqueous solution, but when bound to negatively charged lipids (such as phospholipids on cell membranes), it can form α-helical structures or be rich in β-folded structures [71]. When α-syn is overexpressed or misfolded due to genetic mutations or protein modifications, α-syn fibers may accumulate and aggregate into soluble oligomers and protofibrils, which may eventually stabilize into insoluble amyloid-like fibrillar structures. This misfolding increases the tendency of these proteins to aggregate with other misfolded proteins, leading to fibrillation, aggregate formation, and protein and organelle isolation, ultimately leading to the formation of LBs. Moreover, α-Syn fibers have been shown to be infectious pathogenic factors that spread in a prion-like manner, leading to enhanced aggregation [72].

The main mechanisms responsible for the degradation of misfolded α-syn at synapses are the ubiquitin‒proteasome pathway (UPP) and the autophagy‒lysosome pathway (ALP). UPP is essential for degrading misfolded or damaged proteins within cells. Proteins are tagged with ubiquitin, which targets them for destruction by the proteasome, thus sustaining protein balance. ALP is vital for cellular waste management. It engulfs and degrades cellular debris and damaged organelles through autophagosomes that merge with lysosomes, where they are broken down by lysosomal enzymes, ensuring cell health and functionality. However, the gradual accumulation of α-syn at synapses leads to the breakdown of these systems. Consequently, proteostasis dysregulation in dopaminergic neurons precipitates the accumulation of α-syn, which subsequently impairs protein degradation pathways, thereby establishing a deleterious feedback loop [73,74]. In addition, α-syn aggregation triggers endoplasmic reticulum stress and unfolded protein response (UPR) activation, further driving the development of neurodegenerative diseases [75,76]. Currently, therapeutic strategies for α-syn mainly include two aspects: one is to reduce its abnormal aggregation by regulating the expression and metabolism of α-syn, and the other is to prevent the damage of α-syn to the nervous system by intervening in the formation and stability of α-syn aggregates.

EGCG has been shown to inhibit α-syn aggregation *in vitro*. However, its therapeutic efficacy in PD is hindered by the BBB and strong binding affinities between EGCG and membrane proteins, impeding its accumulation within dopaminergic neurons. To overcome these challenges, researchers have developed multifunctional lipid micelles that contain EGCG. Micelles have been engineered to enhance the transport of EGCG across the blood–brain barrier by utilizing B6, a peptide with a high affinity for the TfR. Additionally, ligands such as mazindol have been employed to selectively target dopaminergic neurons by interacting with the DAT, facilitating its internalization. The results demonstrated a significant increase in the accumulation of EGCG within PD lesions. Furthermore, a remarkable inhibition of α-syn aggregation was observed, accompanied by a notable increase in the population of dopaminergic neurons [77]. Moreover, Kuo et al. conducted a study in which liposomes were surface-modified with Lep and loaded with RES and EGCG. This formulation exhibited neuroprotective effects by downregulating the expression of apoptotic proteins, such as the Bcl-2-associated X protein and α-syn, while upregulating the expression of the antiapoptotic protein B-cell lymphoma 2, TH, and DAT [78]. Similarly, in addition to EGCG, curcumin has the potential to clear α-syn aggregates and treat PD, but it is limited by the BBB, resulting in low bioavailability and poor penetration. Zhang et al. [79] developed a curcumin-based NP system named Curcumin Polyethylene Glycol 8000 Oleate (CPC NPs) modified with polyethylene glycol 8000 oleate, which utilizes the ultrasound-targeted microbubble disruption technique to increase the permeability of the BBB and achieve localized delivery of curcumin to target nuclei in the brains of PD patients. Furthermore, Sela et al. employed transferrin-modified brain-targeted liposomes to accurately deliver the monoclonal antibody Syno4 to dopaminergic neurons in the brains of mice with PD. This approach successfully reduced α-syn aggregation and neuroinflammation, resulting in improved motor function [80]. In addition to being nanocarriers, NPs themselves can be used as therapeutic agents. Aliakbari et al. [81] showed that zwitterionic NLPs with cholesterol and PEG (NLP-Chol-PEG) can effectively inhibit α-syn fibrillization and reduce neurotoxicity and ROS production without altering neuronal intracellular calcium. The results showed that the clearance of α-syn led to the restoration of normal motor behavior, DA levels, and TH expression.

#### 5.2.3 Oxidative stress, mitochondrial dysfunction, and neuroinflammation

The pathogenesis of PD involves the complex interplay of oxidative stress, mitochondrial dysfunction, and neuroinflammation. Oxidative stress refers to the imbalance between oxidation and antioxidant processes within the body and plays an important role in the pathogenesis of PD [82,83]. Under normal circumstances, the body’s oxidation system and antioxidant system maintain a dynamic balance, and free radicals can be promptly eliminated by substances such as catalase, superoxide dismutase (SOD), and glutathione peroxidase. However, oxidative stress occurs when external factors induce a large accumulation of free radicals or a decrease in antioxidant levels. Research on early-stage PD patients has shown significant increases in oxidative damage markers such as malondialdehyde and lipid peroxidation in the substantia nigra. SOD activity and zinc levels are elevated, while glutathione (GSH) is reduced by 40%. The activities of catalase and GSH reductase are also decreased, indicating the occurrence of oxidative stress in PD patients [84]. As a result, the imbalance between oxidation and antioxidant processes in PD leads to various damaging effects, including DNA breakage, lipid autooxidation, protein inactivation, calcium imbalance, and mitochondrial dysfunction. ROS directly damage DA, which is released into the extracellular space by dopaminergic neurons and promotes the production of a substantial number of intracellular oxygen radicals. This leads to the activation of NF-κB and the subsequent release of significant amounts of inflammatory factors. The accumulation of these factors activates nitric oxide synthase, culminating in the production of a large quantity of nitric oxide (NO), which exerts potent toxic effects on dopaminergic neurons.

In PD, oxygen radicals interact with calcium ions, disrupting cellular calcium homeostasis and leading to elevated intracellular calcium concentrations. This disruption initiates a cascade of enzymatic activations involving nicotinamide adenine dinucleotide phosphate oxidase, fatty acid oxidase, and mitochondrial enzymes, which in turn increase the production of oxygen radicals [85]. This interaction establishes a detrimental feedback loop that accelerates dopaminergic neuron loss. Moreover, elevated levels of calcium and oxygen radicals within cells trigger various cell death pathways, including the mitochondrial pathway and apoptosis receptor pathway, further contributing to the rapid degeneration of dopaminergic neurons. Iron ions can react with ROS to produce highly reactive free radicals, leading to oxidative damage and cell death. Additionally, iron ions can increase mitochondrial ROS levels by promoting the production of mitochondrial complex I. Furthermore, iron ions can suppress the cellular antioxidant system, further increasing ROS accumulation [86]. These interactions may exacerbate oxidative stress in PD patients, ultimately leading to neuronal cell death and disease progression.

Mitochondria, essential organelles within cells, primarily function to generate ATP, serving as the primary energy source for cellular activities. This process involves the transfer of electrons from Complex I to Complex IV of the electron transport chain (ETC) and is associated with oxidative phosphorylation. A significant byproduct of mitochondrial ETC activity is ROS, and mitochondrial dysfunction leads to an overproduction of ROS [87]. Importantly, ROS can exert deleterious effects on the ETC itself. In the context of PD, mitochondrial dysfunction plays a pivotal role. It has been demonstrated that, upon metabolism in the brain to 1-methyl-4-phenylpyridinium (MPP+), MPTP selectively inhibits mitochondrial complex I, resulting in the death of dopaminergic neurons in the substantia nigra [88]. Notably, PD patients exhibit a decrease in mitochondrial complex I activity and a decrease in the expression of genes encoding mitochondrial proteins. Certain gene mutations, including those in LRRK2, PRKN, DJ-1, PINK1, FBXO7, and ATP13A2, may precipitate mitochondrial impairment. Furthermore, mitochondrial dysfunction is implicated in processes such as mitochondrial biogenesis, fusion/fission, and mitophagy [89]. In addition to their role as energy suppliers, mitochondria have emerged as central signaling hubs in the regulation of networks associated with innate immunity and inflammation. In response to diverse stressors, mtDNA can translocate to intra- or extracellular compartments. Released mtDNA from mitochondria can serve as damage-associated molecular patterns, stimulating inflammation through interactions with pattern recognition receptors.

Microglia, the resident macrophages of the CNS, maintain cerebral homeostasis and respond to injury or pathogens. In PD, microglia can be activated by a myriad of factors, encompassing abnormal α-syn aggregation, cell damage signals, and environmental factors. Upon activation, microglia release inflammatory mediators, including cytokines, chemokines, and ROS, promoting neurodegeneration. Concurrently, microglia-mediated inflammatory responses can exacerbate oxidative stress. In PD patients, there is evidence of microglial activation in the substantia nigra and elevated proinflammatory factor levels in the brain and CSF [90]. Moreover, dying neurons release oxidized lipids, proteins, and DNA, which in turn activate microglia, perpetuating a vicious cycle of neurotoxicity [91].

Research has shown that enhancing the activity of the antioxidant system and boosting intracellular antioxidant capacity can mitigate oxidative stress caused by ROS. This alleviation of oxidative stress can provide protection against PD. Consequently, antioxidants are viewed as potential therapeutic agents for treating this disease. At present, natural antioxidant compounds from plants are widely utilized to mitigate toxicity, repair the CNS, and prevent the onset of PD. Commonly used natural antioxidants, including curcumin, RES, ginsenosides, quercetin, catechins, *Mucuna pruriens*, *Withania somnifera*, and ursolic acid, play important roles in the prevention and treatment of PD [92,93,94]. However, the effectiveness of these agents is limited by their ability to cross the BBB, which affects their utilization, stability, and solubility at target sites in the brain. To overcome these limitations, the nanosization of plant bioactive compounds for the treatment of PD can maximize therapeutic efficacy, improve the ability of these compounds to penetrate the brain, and enhance stability.

Nanodelivery technologies, such as SLNs, NLCs, NLPs, and nanobubbles, enable the controlled delivery of nanosized bioactive compounds to the brain. For instance, the oral administration of RES encapsulated in liposomes significantly mitigates abnormal rotational behavior, curtails the loss and apoptosis of cells in the substantia nigra, diminishes the levels of ROS, and bolsters the total antioxidant capacity within the substantia nigra tissue. *In vivo* studies have demonstrated that RES encapsulated in liposomes has stronger effects than free RES [95]. Furthermore, incorporating RES into vitamin E nanoemulsions has been found to amplify its pharmacological activity. Pharmacokinetic studies revealed that drug concentrations in nanoemulsions are elevated, consistent with increased antioxidant activity [96]. Additionally, liposomes can codeliver drugs encapsulating RES and EGCG in Lep-modified liposomes and adding SH-SY5Y cells treated with MPP+ can reduce apoptosis-promoting protein Bcl-2-associated X protein and α-syn, and increase apoptosis-inhibiting protein B-cell lymphoma 2, TH, and DAT, thereby enhancing the neuroprotective effect of neurons [78].

Researchers have prepared nanobubbles loaded with curcumin using a mixed nanostructure based on lipids and PLGA via double emulsion evaporation. These nanobubbles can open the BBB and accumulate in the brain when stimulated by low-intensity FUS. Furthermore, they can release curcumin into the CNS, thereby improving motor function in animal models of PD [97]. In addition to natural antioxidants, coenzyme Q10 and vitamin E are commonly used antioxidant molecules in PD treatment. However, coenzyme Q10 has poor solubility, resulting in low oral bioavailability. To address this issue, a nanoemulsion loaded with coenzyme Q10 was developed. When orally administered to rats with PD, this nanoemulsion effectively reduced DA depletion, increased GSH content, and decreased sulfo-barbituric acid reactive substances [98]. In PD rats, the intranasal delivery of a grapefruit flavonoid nanoemulsion loaded with vitamin E can increase the levels of SOD and GSH induced by 6-hydroxydopamine (6-OHDA) while improving motor ability [99]. Furthermore, several drugs are also used to alleviate oxidative stress and neuroinflammation. Polysorbate 80 surface-modified SLNs from formoterol can suppress the expression of the SNCA gene, mitochondrial membrane damage, and oxidative stress markers in a mouse model of PD [100].

In addition to the liposomal delivery of natural antioxidants, other nanomaterials, such as polymer NPs, exosomes, and metal NPs, are widely used to deliver various antioxidants. Some metal NPs can directly alleviate oxidative stress by acting as scavengers for ROS and NO, mimicking the function of major antioxidant enzymes involved in oxidative stress, including metal oxides and nanozymes that mimic peroxidase, SOD, and other antioxidant enzymes. In summary, the abovementioned research indicates that nanosization techniques can enhance the penetration, stability, and efficacy of natural antioxidants in the brain, suggesting that they are promising therapeutic strategies for PD. However, further research and clinical trials are required to validate the safety and effectiveness of these agents.

#### 5.2.4 NTFs

NTFs are a group of small extracellular proteins that regulate the survival, development, and function of neurons in the nervous system. These genes belong to four families: the nerve growth factor (NGF) family (also known as neurotrophins), the glial cell line-derived neurotrophic factor (GDNF) family, the cerebral dopamine neurotrophic factor (CDNF)/mesencephalic astrocyte-derived NTF family, and other NTFs. Through binding to specific receptors, NTFs regulate multiple aspects of neuronal survival, development, function, and synaptic connectivity, playing crucial roles in normal development and maintenance of the nervous system. Additionally, some NTFs have anti-inflammatory, antiapoptotic, remyelination, and axonal regeneration properties, and they promote tissue repair and regeneration by facilitating the involvement of adult stem cells in tissue repair after neural damage [101,102,103].

In the context of PD, changes in NTFs have been observed in both clinical and experimental studies. Research has shown a significant decrease in NTFs, such as those in the SNpC and basal ganglia, in regions associated with dopaminergic neurodegeneration in PD patients [104]. One of the well-studied NTFs in PD is brain-derived neurotrophic factor (BDNF), which is highly expressed in motor-related areas of the brain. Reductions in BDNF levels, which are closely related to dopaminergic neurodegeneration, have been observed in the substantia nigra and putamen of PD patients. Moreover, the overexpression of α-syn in certain brain regions of PD patients is associated with the downregulation of BDNF [105]. Another important NTF in the maintenance of the dopaminergic system is GDNF. GDNF is highly expressed in brain regions involved in motor control in the human brain [106]. However, the decreases in GDNF in the putamen, substantia nigra, and motor control areas of the brain in PD patients are more significant than those in BDNF patients.

NTFs play a critical role in the nervous system, especially in the treatment of PD. However, different methods of administering NTFs in clinical trials have not yielded the expected results and have resulted in severe side effects. The delivery and selectivity of NTFs play crucial roles in clinical cases of PD. Systemic administration of NTFs has poor pharmacokinetic effects and results in very low permeability through the BBB; thus, expensive and risky intracranial surgery is needed to directly deliver NTFs into the brains of PD patients. Only patients in the middle and late stages of the disease can undergo surgical treatment. In recent years, NP-based strategies have received widespread attention for their ability to improve the stability and delivery of NTFs to the brain. This strategy can enhance the bioavailability and stability of NTFs, reduce dosage and side effects and is expected to become a new approach for future PD treatments.

Xia et al. developed a Trojan horse liposome (THL) by intravenous injection of GDNF plasmid DNA targeting the TfR in rats. They observed an increase in the GDNF concentration in the substantia nigra, which led to a reduction in apomorphine-induced rotation at different doses and an increase in TH activity in the striatum [107]. Similarly, the intranasal delivery of GDNF via liposomes to PD rats increases brain GDNF levels and has a neuroprotective effect [108]. In addition to liposomes, NLCs have also been investigated as useful DDSs for the gold standard, NTF-GDNF, in PD treatment. In fact, chitosan-modified GDNF-loaded NLCs were nasally administered to a PD rat model. Chronic administration of these chitosan-modified lipid NPs successfully delivered GDNF to 6-OHDA-lesioned rats for 2 weeks, resulting in the restoration of motor symptoms [109]. Although GDNF is one of the most studied NTFs for the treatment of PD, other similar molecules have also been encapsulated to improve their bioavailability. Collagen nanocrystal carriers encapsulating bFGF have been studied as a novel method of nasal administration. Nasal administration of NLCs improved rotational behavior, monoamine neurotransmitter levels, and TH expression in *in vitro* and *in vivo* studies without adverse effects on nasal mucosal integrity [110]. Niu et al. [111] discovered that the use of FUS and liposome delivery of small ubiquitin-related modifier fused with fibroblast growth factor 20 (FGF20) improved motor behavior and protected dopaminergic neurons in a rat model of PD.

In conclusion, the delivery of NTFs using NP systems has emerged as a new research hotspot in the treatment of PD. By utilizing NPs for NTF delivery, their bioavailability and stability can be enhanced while reducing their dosage and side effects. Although further research is still needed to improve the effectiveness of this treatment approach, it has already demonstrated significant potential and is expected to become a key technology in future PD therapy.

#### 5.2.5 Gene therapy

Recent gene engineering technologies have led to the emergence of gene therapy as a promising approach for treating CNS diseases. Compared to traditional small molecule-based therapies, gene therapy offers several advantages, such as the modulation of protein-coding genes beyond the reach of conventional small molecules, high target specificity, reversible effects, and potential reprogramming without altering drug pharmacokinetics [112,113]. Genotype analysis of various CNS diseases has revealed novel therapeutic targets, and several promising treatment strategies aimed at activating or silencing these targets, including plasmid DNA, small interfering RNA (siRNA), antisense oligonucleotides, miRNAs, and mRNAs, have been identified.

Gene therapy for CNS diseases is limited by the restrictive nature of the BBB and blood-cerebrospinal fluid barrier (BCSFB), which play crucial roles in maintaining CNS homeostasis and preventing the entry of harmful compounds. Various approaches have been explored to enhance drug delivery into the brain, including direct CNS administration, BBB disruption, and carrier-mediated delivery. However, these methods have limitations and potential adverse effects. To address these challenges, nanomaterials have emerged as promising vehicles for nucleic acid delivery, offering improved stability, bioavailability, and penetration across the BBB and BCSFB. Nanomaterials such as liposomes, polymers, inorganic NPs, and extracellular vesicles can be tailored for precise targeting of CNS disease sites.

Several nonviral gene delivery systems, such as liposomes, have been reported for use in PD treatment. The RVG-decorated liposomes exhibited suitable properties for potential *in vivo* applications and successfully induced α-syn gene silencing in primary neurons without affecting cell viability [114]. The authors employed liposomal delivery of siRNA targeting the GluN1 subunit of NMDA receptors and demonstrated its effective and selective reduction of synaptic NMDA receptor currents compared to synaptic α-amino-3-hydroxy-5-methyl-4-isoxazole-propionic acid receptor currents [115]. TH is an important enzyme involved in the synthesis of DA in the body. Therefore, increasing the expression or activity of TH can enhance DA synthesis and potentially alleviate symptoms of PD. A study demonstrated that in the 6-OHDA rat model of PD, targeted immunoliposomes loaded with plasmids expressing TH and the TfR Mab resulted in complete normalization of TH activity in the striatum [116].

Lipid NPs have changed the history of COVID-19 vaccines through the use of mRNAs. Restoring the numbers and functions of regulatory T cells (Tregs) represents a novel therapeutic strategy for treating neurodegenerative disorders. The authors developed a novel LNP containing mRNA encoding granulocyte-macrophage colony-stimulating factor (Gm-csf mRNA), which can increase plasma GM-CSF levels and peripheral CD4+CD25+FoxP3+ Treg cell populations in PD rats [117]. Neuroinflammation has been noted in PD, resulting in neuronal damage and death that accelerate the progression of PD. Several studies have shown that the level of l-PGDS in the CSF of PD patients is associated with clinical symptoms and disease progression. The use of mannosylated LNPs for the targeted delivery of l-PGDS ASO to brain border-associated macrophages in rats leads to the silencing of neuroinflammation-related genes and represents a significant step forward in the development of gene therapy platforms for the advanced treatment of neuroinflammation-related pathologies using ASO@LNP nanovectors [118]. Glial cell line-a (GDNF) is a potent agent for PD therapy due to its neuroprotective and neurotrophic effects. Xia et al. utilized THLs targeted with a Mab to rat TfR to deliver intravenous GDNF plasmid DNA. Elevated concentrations of GDNF in the substantia nigra led to a dose-dependent reduction in apomorphine-induced rotations and an increase in striatal TH enzyme activity [70]. Similarly, the delivery of the GDNF plasmid using THLs targeted to TfRs can eliminate neurotoxic effects through multiple intravenous administrations [119]. FUS induced by microbubbles is a promising technique for noninvasive opening of the BBB to allow targeted delivery of therapeutic substances into the brain and thus noninvasive delivery of genetic vectors for CNS treatment. The use of FUS to deliver liposome-coupled microbubbles to mediate the gene transfer of the GDNF plasmid into the brain mitigates behavioral deficits and neuron loss in a rat model of PD [120,121].

#### 5.2.6 Stem cell therapy

In the twenty-first century, the significance of stem cells in medical research and therapeutic applications has dramatically increased. Stem cell therapy represents a therapeutic approach wherein appropriate cells or tissue groups are transplanted into the host brain to substitute for neurons and brain tissues damaged by neurodegenerative diseases, thereby reconstructing or restoring neural function. Stem cells are characterized by unique features, including the capacity for self-renewal and differentiation into various cell types [122]. They maintain an undifferentiated state (pluripotency) and can potentially differentiate into multiple mature cell types [123]. Depending on their origin, stem cells can be categorized as embryonic stem cells, mesenchymal stem cells (MSCs), adult stem cells, or induced pluripotent stem cells (iPSCs).

Stem cell therapy offers a promising treatment approach for neurodegenerative diseases, including PD. In PD, stem cell therapy aims to promote the differentiation of stem cells into DA-producing neurons, which are subsequently delivered to damaged areas of the patient’s brain to restore neural function and alleviate the progression of the disease. Injection of MSCs from different sources into PD rats effectively improved neurological function, promoted the survival of damaged neurons, and increased DA levels [124,125,126]. In clinical trials, MSCs from different sources, administered through intracerebral transplantation, arterial injection, or venous injection, have been found to improve unified parkinson’s disease rating scale scores in PD patients [127]. Moreover, both human embryonic stem cells (hESCs) and iPSCs have distinctive characteristics that render them suitable for investigating the progression of PD. These cells have been implanted into model organisms under established PD conditions, yielding a combination of results that are both varied and encouraging.

Although stem cell therapy holds great promise for treating neurodegenerative diseases, there are still several limitations. For instance, the selection of appropriate cell types is crucial for treating CNS diseases. The presence of the BBB makes it difficult for cells to be delivered into the brain. Additionally, transplanted cells may lead to immune reactions, tumor formation, or inappropriate cell proliferation, thereby increasing treatment risk. The efficiency of stem cell differentiation and the functional expression of differentiated cells are also challenging. The survival rate of transplanted cells in the body is an important concern. Furthermore, monitoring and tracking transplanted stem cells in the human body are difficult. However, nanotechnology combined with stem cell therapy can overcome the abovementioned difficulties to a certain extent. For example, functionalized nanocarriers can target the delivery of stem cells and drugs to the lesion area, improving treatment efficacy and reducing the impact on healthy tissues. These carriers can protect stem cells, facilitate their passage through the BBB, and increase their survival rate after transplantation. Nanotechnology-delivered biomolecules can precisely control stem cell differentiation and reduce immune rejection. Nanocarriers can track and monitor the behavior of stem cells to assess treatment effects. Furthermore, nanoscaffolds provide an ideal microenvironment for stem cells, aiding in the repair of neural tissues. Nanocarriers also increase the bioavailability of drugs, reducing dosage and side effects, thus opening up new avenues for the treatment of CNS diseases.

In recent years, researchers have been continuously exploring the use of lipid-based nanocarriers for targeted differentiation and treatment of stem cells. Chabra et al. reported that SLNs can maintain the characteristics of mouse MSCs by serving as a scaffold for both stem cell attachment and stemness while also inducing stem cell differentiation [128]. Similarly, Kuo et al. designed a heparinized cationic SLN loaded with NGF that can direct the differentiation of stem cells into neurons [129]. The same team also prepared amphiphilic solid lipid nanoparticles (ASLNs) with the Ln5-P4 peptide for loading NGF and retinoic acid. This nanocarrier can bind to α3β1 integrin on iPSCs and promote their differentiation into neurons. These findings indicate that Ln5-P4/NGF-RA-ASLNs represent a potential system for inducing iPSC-derived neuron differentiation, providing a new approach for treating neurodegenerative diseases [130]. Takeda et al. successfully delivered miRNA to human mesenchymal stem cells (hMSCs) using synthetic bioreducible lipids, and miRNA-9 promoted their neural differentiation, demonstrating the potential of these lipids in gene delivery and stem cell programming [131]. Similarly, Zhao et al. developed a novel cationic lipidoid-based nanocarrier to deliver Cas9 mRNA and sgRNA targeting the neuronal restrictive silencing factor to induce neural-like cell differentiation in hMSCs [132]. These studies demonstrate the potential application value of lipid-based nanocarriers in stem cell therapy and differentiation, providing a solid foundation for the treatment of neurodegenerative diseases, including PD.

## 6 Discussion

The occurrence of PD is associated with complex pathogenic mechanisms, and aging is one of the primary reasons for the global increase in PD cases. Current treatment strategies rely on NTFs, antioxidants, and DA replacement medications conducted in various cellular or animal models [133]. However, these molecules often struggle to cross the BBB, which limits their effectiveness and clinical application. To overcome these obstacles, lipid-based nanocarrier DDSs have shown promise. These systems exhibit minimal toxicity, enhance drug loading, provide sustained release, and safely traverse the BBB. Lipid carriers can also be conjugated with genes, peptides, and ligands to facilitate the active penetration of drugs through the BBB and to target therapeutic agents to the affected sites. The FDA’s approval of the Spikevax^®^ and Comirnaty vaccines for COVID-19 immunization marks a milestone that may propel research into lipid-based nanomedicines, promoting additional clinical trials and the development of commercial products. For the mechanisms of oxidative stress, mitochondrial damage, inflammation, and misfolding of the α-syn protein in PD, lipid NPs have been developed to deliver various molecules and drugs to alleviate oxidative stress, reduce neuroinflammation, eliminate neurotoxic α-syn, and promote the regeneration of dopaminergic neurons as therapeutic strategies.

Despite the significant potential of lipid-based NPs for PD demonstrated in preclinical studies, their clinical application remains limited. Challenges include the potential accumulation of NPs in the liver, the formation of a protein corona through binding with serum proteins, and the possibility of triggering immune responses. To address these challenges, the development of nanotherapeutics that are both selective and biocompatible is possible through the optimization of NP surfaces. However, ongoing research into the molecular neuropathology of PD, as well as the advancement of nanocomposite delivery systems and the refinement of surface properties, is needed to ensure precise targeting and biocompatibility. All abbreviations are listed in Table 3.

**Table 3:** Abbreviations

Abbreviations	Full Names	Abbreviations	Full Names
6-OHDA	6-hydroxydopamine	LDLR	Low-density Lipoprotein Receptor
AAV	Adeno-associated Virus	L-DOPA	L-3,4-dihydroxyphenylalanine
ABCB1	ATP-binding Cassette sub-family B member 1	LEPR	Leptin Receptor
ALP	Autophagy-Lysosome Pathway	LNP	Lipid Nanoparticle
AMT	Adsorptive-Mediated Transcytosis	LRRK2	Leucine-Rich Repeat Kinase 2
APP	Amyloid Precursor Protein	MANF	Mesencephalic Astrocyte-derived Neurotrophic Factor
BBB	Blood Brain Barrier	MAO-B	Monoamine Oxidase-B
BDNF	Brain-derived Neurotrophic Factor	MPP+	1-methyl-4-phenypyridinium
BSCB	Blood-Spinal Cord Barrier	MPTP	1-methyl-4-phenyl-1,2,3,6-tetrahydropyridine
CDNF	Cerebral Dopamine Neurotrophic Factor	MSC	Mesenchymal Stem Cell
CMT	Endogenous-Carrier-Mediated Transport	mtDNA	Mitochondrial DNA
CNS	Central Nervous System	NGF	Nerve Growth Factor
COMT	Catechol-O-methyltransferase	NLC	nanostructured lipid carriers
CSF	Cerebrospinal Fluid	NO	Nitric Oxide
DA	Dopamine	NP	Nanoparticles
DAT	Dopamine Transporter	NTF	Neurotrophic Factor
DBS	Deep Brain Stimulation	PARK7	Parkinson Disease Protein 7
EC	Endothelial Cell	PD	Parkinson’s Disease
EGCG	Epigallocatechin Gallate	PEG	Polyethylene Glycol
ER	Endoplasmic Reticulum	P-gp	P-glycoprotein
ETC	Electron Transport Chain	PINK1	Putative Kinase Protein 1
FcRN	Neonatal Fc Receptor	PINK1	PTEN-Induced Putative Kinase 1
GDNF	Glial Cell Line-derived Neurotrophic Factor	PRKN	Parkin RBR E3 Ubiquitin Protein Ligase
GSH	Glutathione	RMT	Receptor-Mediated Transport
H2O2	Hydrogen Peroxide	RNS	Reactive Nitrogen Species
hESCs	Human Embryonic Stem Cells	LDLR	Low-density Lipoprotein Receptor
IGFR	Insulin-like Growth Factor Receptor	L-DOPA	L-3,4-dihydroxyphenylalanine
iPSCs	Induced Pluripotent Stem Cells	LEPR	Leptin Receptor
IR	Insulin Receptor	LNP	Lipid Nanoparticle
LB	Lewy Body	LRRK2	Leucine-Rich Repeat Kinase 2
LDL	Low-Density Lipoprotein	MANF	Mesencephalic Astrocyte-derived Neurotrophic Factor
